# Gut–lung axis in asthma and obesity: role of the gut microbiome

**DOI:** 10.3389/falgy.2025.1618466

**Published:** 2025-06-16

**Authors:** Hiroki Tashiro, Yuki Kuwahara, Koichiro Takahashi

**Affiliations:** Division of Hematology, Respiratory Medicine and Oncology, Department of Internal Medicine, Faculty of Medicine, Saga University, Saga, Japan

**Keywords:** asthma, obesity, gut microbiome, short-chain fatty acid (SCFA), airway inflammation

## Abstract

Asthma is a heterogeneous disease whose severity is exacerbated by obesity. Despite its clinical importance, targeted therapies for asthma in obese patients remain limited. Recent evidence highlights the gut microbiome as a crucial factor linking metabolic and immune pathways involved in both asthma and obesity. This review explores the complex interplay between the gut microbiome, immune responses, and the gut–lung axis, emphasizing how microbial composition, diversity, and metabolites, such as short-chain fatty acids (SCFAs), influence airway hyperresponsiveness (AHR) and airway inflammation. Obesity alters the gut microbiome, contributing to systemic inflammation and metabolic dysfunction. Furthermore, asthma phenotypes related to obesity are associated with specific gut microbial profiles, suggesting a causal relationship. Animal studies have demonstrated that manipulation of the gut microbiome through diet, antibiotics, or microbial transplantation can alter asthma outcomes, particularly in obesity models. Given these findings, targeting the gut microbiome might be a promising therapeutic strategy for asthma in obese individuals. Potential interventions include probiotics, prebiotics and antibiotics, all of which have shown varying degrees of effectiveness in modulating airway inflammation and reducing asthma severity. This review provides a comprehensive overview of current knowledge and proposes future directions for microbiome-targeted therapies in managing severe asthma associated with obesity.

## Introduction

Asthma is a heterogeneous disease, and obesity is one of the important comorbidities of asthma, which enhances disease severity by inducing excessive airway hyperresponsiveness (AHR) and airway inflammation (1). Unfortunately, specific treatments for severe asthma with obesity are not available, with body weight reduction being the only recommended treatment (2). Recently, the gut microbiome has been highlighted for its role in regulating not only local gastrointestinal diseases, including inflammatory bowel diseases, but also systemic diseases, including asthma (3). Additionally, increased evidence has revealed that obesity itself and obesity-induced diseases, such as cardiovascular diseases, diabetes, and metabolic syndrome, are mechanistically associated with the gut microbiome (4). Based on these data, it is plausible to suggest a close relationship between the gut microbiome and severity of asthma in obese individuals. Furthermore, the gut microbiome might serve as a specific therapeutic target for the treatment of asthma with obesity. In this review, we present evidence for the interaction between the gut microbiome and obesity, asthma, and asthma with obesity, respectively, especially focusing on the immune modulatory effect as the underlying mechanism. In the final part of this review, we explore the potential utility of new therapeutic strategies of gut microbiome-targeted treatment for severe asthma in obese individuals. This narrative review is based on a literature search conducted using PubMed and Google Scholar. We included studies published from January 2010 to April 2024, written in English. Search keywords included *asthma*, *obesity*, *gut microbiome*, *short-chain fatty acids*, *airway inflammation*, and *animal models*. Both original research articles and reviews were considered. We prioritized studies based on their relevance to the topic, mechanistic insight, citation frequency, and methodological quality. While no formal quality scoring system was applied, each article was evaluated for scientific rigor and clarity of findings.

### Overview of the gut microbiome

The gut microbiome, which consists of trillions of microorganisms, such as bacteria, viruses, and fungi, has been increasingly recognized as an external organ of the human body (3, 5). Several studies have indicated that gut microbiome composition, diversity, and metabolites, such as fatty acids, differ between healthy and diseased individuals (6, 7), indicating that the gut microbiome impacts human health. For example, the characteristics and diversity of the gut microbiome are altered by aging (8), and a youth-related pattern of the gut microbiome, such as *Bacteroides*-dominant enterotype, is associated with longevity in humans (9). Additionally, the main metabolites of the gut microbiome, including bile acids, short-chain fatty acids (SCFAs), trimethylamine N-oxide, and derivatives of tryptophan, are potentially involved in human and animal longevity (10, 11). Along with individual variability in the characteristics of microorganisms in the gut, external factors, such as antibiotic usage, early life events, and diet, also have a strong impact on human health through their gut microbiome (12–14). The vast dataset of human metagenomic assays suggests that antimicrobial consumption influences an individual's gut microbiome, with increased prevalence of antimicrobial resistance genes (15). In mice, antibiotic administration resulted in variable perturbation of the gut microbiome, as assessed by 16s ribosomal RNA sequencing analysis, depending on the type of antibiotic, such as ampicillin, metronidazole, neomycin, or vancomycin (16). Another study involving a longitudinal analysis of stool samples from 903 children revealed that receipt of breast milk, either exclusively or partially, as an early life event, had the highest association with the microbiome structure, with a higher level of *Bifidobacterium* species (13). A recent human cohort of 21,561 individuals showed variability in microbial profile depending on the individual's dietary pattern, whether omnivorous, vegetarian, or vegan, with interaction with their health outcomes in terms of favorable cardiometabolic markers (17). We previously reported that a high-fiber diet and high-fat diet drastically changed the gut microbiome compared to normal chow in mice (18–20). These data suggested that the gut microbiome impacts human health, and should be taken into consideration in the mechanistic analysis of health problems, including obesity and asthma (1, 21).

### Interaction between the gut microbiome and immunity

The gut is the largest organ related to the mammalian immune system, containing more than 70% of the immune cells of the entire body. Additionally, the gut microbiome influences the immune system, including intestinal and extra-intestinal organs (22, 23). The gut microbiome exerts functional effects on the immune system in both healthy and diseased individuals, likely through the barrier function of the mucus layer that is regulated by immunoglobulin A (IgA), and by direct activation of the immune system through gut-associated lymphoid tissue (GALT) and immune cells (22, 24). The mucus layer in the intestine physically and functionally serves as a barrier between harmful external molecules and microorganisms and the inside of the human body (25). In this process, secretory IgA is recognized as the first line of defense for maintaining gut homeostasis, with increasing evidence of the role of the gut microbiome in regulating this system (26). Nakajima A et al. reported that IgA production in mucus is increased by a specific bacterium, namely *Bacteroides* species, and it is involved in the diversity and metabolic activity of the gut microbiome, and consequently in gut homeostasis (27). Thereafter, immune cells, such as T cells and B cells in GALT, serve as downstream mechanisms in the barrier function of the mucus layer for impact of immunity on gut microbiome (28). GALT consists of multi-follicular lymphoid tissue, such as Peyer's patches and numerous isolated lymphoid follicles, which exist in large numbers in immune cells in the small and large intestines (29). In mice, B cell responses derived from Peyer's patches in GALT were shown to be altered in germ-free mice compared to normal mice free of specific pathogens (30, 31). Other studies have reported that *Bacteroides acidifaciens* and *Prevotella buccalis* induce Peyer's patch-dependent IgA production, which stimulates the mammary gland to secrete IgA in milk (32). Localized and systemic inflammation caused by immune reactions is also affected by the gut microbiome. For example, in inflammatory bowel diseases, a higher proportion of *Bacteroides*, *Bacteroidales*, and *Enterobacteriaceae*, and lower proportion of *Firmicutes*, such as *Faecalibacterium prausnitzii*, induces reduction of SCFAs as metabolites from the bacteria, which exacerbate colitis, characterized by an increase in tumor necrosis factor alpha (TNF-α), interferon gamma (IFN-γ) and interleukin 17A (IL-17A), with differentiation of T helper cell 1 (Th1), Th2, and Th17 from naïve T cells (33). In systemic lupus erythematosus, a systemic inflammatory disease, a higher proportion of *Lachnospiraceae*, *Ruminococcaceae*, and *Rikenellaceae*, and lower proportion of *Lactobacillaceae* induces lupus nephritis by polarized Th17-type inflammation (33). Since obesity itself has the potential to cause systemic inflammation, and gut immunity contributes to innate and acquired systemic immunity, as mentioned above, gut immunity might have the ability to modulate the severity of obesity-induced asthma (33–35).

### Interaction between the gut microbiome and obesity

Increasing evidence has shown that obesity itself, and the pathophysiology of obesity-related diseases, such as diabetes mellitus, cardiovascular diseases, and metabolic syndrome are influenced by the gut microbiome. Along with food intake and dietary habits, many factors are directly associated with gut microbial perturbation and body weight in obese individuals compared to lean individuals. The interaction between the gut microbiome and obesity is far from simple. In 2006, the gut microbiome, along with metabolites such as SCFAs, were compared between genetically induced obese mice, called ob/ob mice, and their lean littermates. In that study, the principal component of the gut microbiome, ratio of *Firmicutes* to *Bacteroidetes* (which was greater in obese mice) and concentration of SCFAs were significantly different between the two groups. Additionally, colonization of the gut microbiome derived from obese mice into germ-free mice induced an increase in body fat, suggesting the presence of an obesity-specific gut microbiome (36). Previous studies in other genetic or diet-induced obese mice models also showed similar results (18, 20). In humans, comparative analysis of the gut microbiome in a large Korean cohort of 1,463 subjects who were categorized based on body mass index (BMI) showed phylogenetic diversity and significant differences in the principal component of the gut microbiome between lean and obese individuals (37). The authors suggested that the mechanisms for these differences could be that the gut microbiome increases the risk factors for obesity, such as induction of energy uptake, fat storage, and appetite, in these individuals (38–40). Obesity also induces metabolic dysfunction, such as insulin resistance and systemic inflammation (4). For example, Takeuchi T et al. reported that in 306 individuals, the pattern of their gut microbiome was associated with either insulin resistance or insulin sensitivity, with distinct patterns of carbohydrate metabolism (41). Additionally, interactions were observed between concentrations of TNF-α and IFN-γ in blood, as indicators of systemic inflammation, and gut microbiome composition in humans, which is associated with microbial metabolic pathways, including palmitoleic acid metabolism and degradation of tryptophan to tryptophol (42). These data provide evidence for the close association between the gut microbiome and obesity. In the treatment of obesity, weight reduction by all methods, dietary restriction, exercise, and bariatric surgery, reportedly impacts the gut microbiome (43). An intervention study using a low energy diet for weight reduction in 211 overweight or obese participants revealed significant alterations in the gut microbiome with weight loss. In particular, greater microbial richness and diversity, along with greater abundance of *Akkermansia* and *Christensenellaceae* R-7 group, and decrease in *Pseudobutyrivibrio*, acetogenic *Blautia*, and *Bifidobacterium* species were seen with weight loss (44). Several studies have also reported that along with weight reduction, bariatric surgeries, such as gastric bypass and sleeve gastrectomy, have the ability to manipulate the gut microbiome (45–47). These data also indicated that the gut microbiome is not only involved as a mechanistic factor in obesity, but also might be a therapeutic target for weight loss.

### Gut microbiome and asthma: the gut–lung axis

The gut microbiome is associated with the development and severity of asthma. It is widely known that the human microbial composition matures within the first few years of life, and is potentially a risk factor for the development of asthma (48, 49). Stokholm J et al. reported that in 690 participants, immature microbial composition at 1 year of age was associated with an increased risk of asthma at the age of 5 years (50). Others also reported that infants at high risk for asthma exhibited delayed maturation of gut microbiome diversification compared to healthy infants, and early intervention with *Lactobacillus rhamnosus GG* showed potential to modify the gut microbiome and host immunity, with elevation of regulatory T cells (Tregs) (51). Indeed, an observational study in 152 children with food allergy showed that differences in *Bacteroides* and *Bifidobacterium* species were associated with higher rates of asthma (52). In mice, ozone, an air pollutant that is a trigger for asthma, induces AHR and inflammation (53, 54), with this phenomenon being attenuated by antibiotics and depletion of the gut microbiome, and in germ-free mice (16). Additionally, ozone exposure causes greater AHR in male mice compared to female mice, with the sex difference being abolished by depletion of the gut microbiome by antibiotics (55). In that report, transplantation of the gut microbiome from male to female mice resulted in greater ozone-induced AHR and airway inflammation in the female recipients, indicating that the gut microbiome directly influences AHR and airway inflammation, both of which are pivotal clinical characteristics of asthma (55). The precise mechanisms linking the gut and lung, called the gut–lung axis, are, however, still unclear, although the role of metabolites of the gut microbiome, such as SCFAs, gut hormones, and immune reactions, including dendritic cells (DCs), Tregs, lymphocytes, and cytokines, have been suggested (56, 57). SCFAs, including acetate, butyrate, and propionate, with carbon chain lengths of C2 to C6, are synthesized by specific microbes upon fermentation of ingested fiber, and have the potential to manipulate the pathophysiology of asthma (58). In children, asthmatic individuals showed significant reduction of fecal butyrate, with lower levels of butyrate-inducing bacteria, such as *Faecalibacterium* and *Roseburia* species in the gut, as compared to healthy volunteers (59). In mice, house dust-induced airway inflammation was attenuated by a high-fiber diet with perturbation of the gut microbiome, likely via the effect of free fatty acid receptor 3, a specific receptor of SCFAs (60). Conversely, we reported that a high-fiber diet induced greater AHR and airway inflammation with manipulation of the gut microbiome and increase in the serum concentration of SCFAs. Reportedly, propionate, a SCFA, induced exacerbation of ozone-induced AHR and airway inflammation (16, 18). Additionally, the signaling of gut hormones, such as glucagon-like peptide 1 (GLP-1) secreted by L cells in the small intestine, is regulated by gut microbiome manipulation (61–64). Importantly, GLP-1 and its receptor signal affect the pathophysiology of asthma. A retrospective cohort study of 4,373 asthmatic patients who were also being treated with diabetic drugs showed that adult patients with asthma treated by a GLP-1 receptor agonist for their type 2 diabetes mellitus had lower rates of asthma exacerbations than those treated with other anti-diabetic drugs (65). In mice, the GLP-1 receptor is expressed more in lung tissue compared to other organs, such as the small intestine, brain, heart, and kidney. Additionally, AHR induced by the inhalation of ovalbumin and lipopolysaccharides was significantly attenuated by treatment with liraglutide, a GLP-1 receptor agonist (66). Accumulating evidence underscores the existence of complex and multi-layered crosstalk between the bidirectional gut–lung axis and the host immune system. In mice, vancomycin, an antibiotic that manipulates the gut microbiome with reduction of SCFAs in the gut, attenuated ovalbumin- and papain-induced airway inflammation. Importantly, migration of DCs was enhanced by treatment with vancomycin, and this phenomenon was restored by supplementation of SCFAs (67). Other researchers also indicated that SCFAs in blood affect various types of DCs, such as monocyte DCs, conventional DCs that deactivate Th2 effector cells and attenuate allergic airway inflammation (56). These data suggest that the gut microbiome and pathophysiology of asthma are closely inter-related, although more detailed, precise data are required to further elucidate this interaction.

### Interaction between asthma and obesity

Increasing evidence has clarified that obesity is associated with the severity of asthma [see (1, 21)]. Although the clinical and biological characteristics of asthma are heterogeneous, previous cluster analysis of a large cohort of asthmatic patients identified various features of asthma with obesity, including late onset asthma, preponderance in females, greater severity of symptoms, low sputum eosinophil counts, less atopy, moderate AHR and reversibility of airway obstruction, and low responsiveness to inhaled corticosteroids (68). Other similar analyses (69, 70) on the clinical characteristics of asthma with obesity also showed frequent exacerbation, type 2 low airway inflammation in adult-onset asthma, decreased pulmonary function, and AHR with a female preponderance (21). Indeed, we previously reported that in a total of 56 patients with adult-onset asthma, being overweight, defined as a BMI greater than 25 kg/m^2^, was associated with a higher annual exacerbation rate and lower blood eosinophil count, characterized as type 2 low airway inflammation (71). Other studies have also reported similar results (72). Additionally, in another of our previous studies, we focused on pulmonary function in 193 patients with asthma and 2,159 patients without asthma, and found that obesity reduced pulmonary function, including forced vital capacity and forced expiratory volume in 1 s, in patients with asthma, but not in those without asthma (73). These data indicate that obesity has specific effects on pulmonary functional decline in patients with asthma, but not in those without asthma. Although the inflammatory phenotype of asthma with obesity is still debatable (74), it might involve an increased incidence of type 2 low airway inflammation, as mentioned above (71). Indeed, in mice, those with obesity caused by diet or genetic factors showed exacerbation of AHR and neutrophilic airway inflammation with increasing expression of IL-17A in the lung (18, 75, 76) and Th17 lymphocytes, suggesting that type 3 innate lymphoid cells might be responsible for worsening the AHR and airway inflammation in such animals (77, 78). As possible severity mechanisms, corticosteroid resistance, cytokines, and fatty acids should be considered. In our retrospective analysis of 56 patients with adult-onset asthma, the annual exacerbation rate in overweight asthma patients was higher than that in normal weight individuals despite the greater use of high doses of inhaled corticosteroids in overweight individuals, suggesting the possibility of corticosteroid resistance in this population (71). Indeed, levels of mitogen-activated protein kinase phosphatase-1, as a glucocorticoid-responsive gene, are significantly lower in obese than in non-obese asthma patients, which also supports this phenomenon (79). In terms of cytokines, IL-17A, which is a strong activator of neutrophils (80), is significantly increased by obesity, and is associated with exacerbation of the pathophysiological factors of asthma in humans and mice (18, 75, 76, 78, 81). IL-6, a predictive marker of systemic inflammation and metabolic syndrome in obesity (82) is also involved in the severity of asthma with obesity. A study from the USA evaluating a severe asthma cohort revealed that the plasma concentration of IL-6 was associated with exacerbation of asthma in obese individuals, and that IL-6 concentration correlated with BMI in this cohort (83). Our biomarker analysis study also indicated that serum concentrations of IL-6 and the annual exacerbation ratio were significantly higher in overweight asthma patients than those who were not overweight (71). In mice, obese mice showed greater neutrophilic airway inflammation with elevation of IL-6 levels in broncho-alveolar lavage (BAL) fluid, which was reduced by neutralizing antibodies for IL-6, indicating that IL-6 also has the potential to mediate obesity-induced asthma severity (84). In terms of fatty acids, long-chain (C12-C22) fatty acids might be affected worse in obese asthma patients. In mice, a high-fat diet consisting of poly saturated fatty acids, such as palmitic acid, induced obesity in mice, with these mice showing greater AHR and neutrophilic airway inflammation following exposure to house dust mites (HDMs) than lean mice. Importantly, systemic administration of palmitic acids exacerbated HDM-induced AHR and neutrophilic airway inflammation (75). Hence, there is a close association between obesity and asthma, although more detailed data are needed to clarify the precise mechanisms for this association.

### Impact of the gut microbiome on asthma with obesity

**Figure 1:**
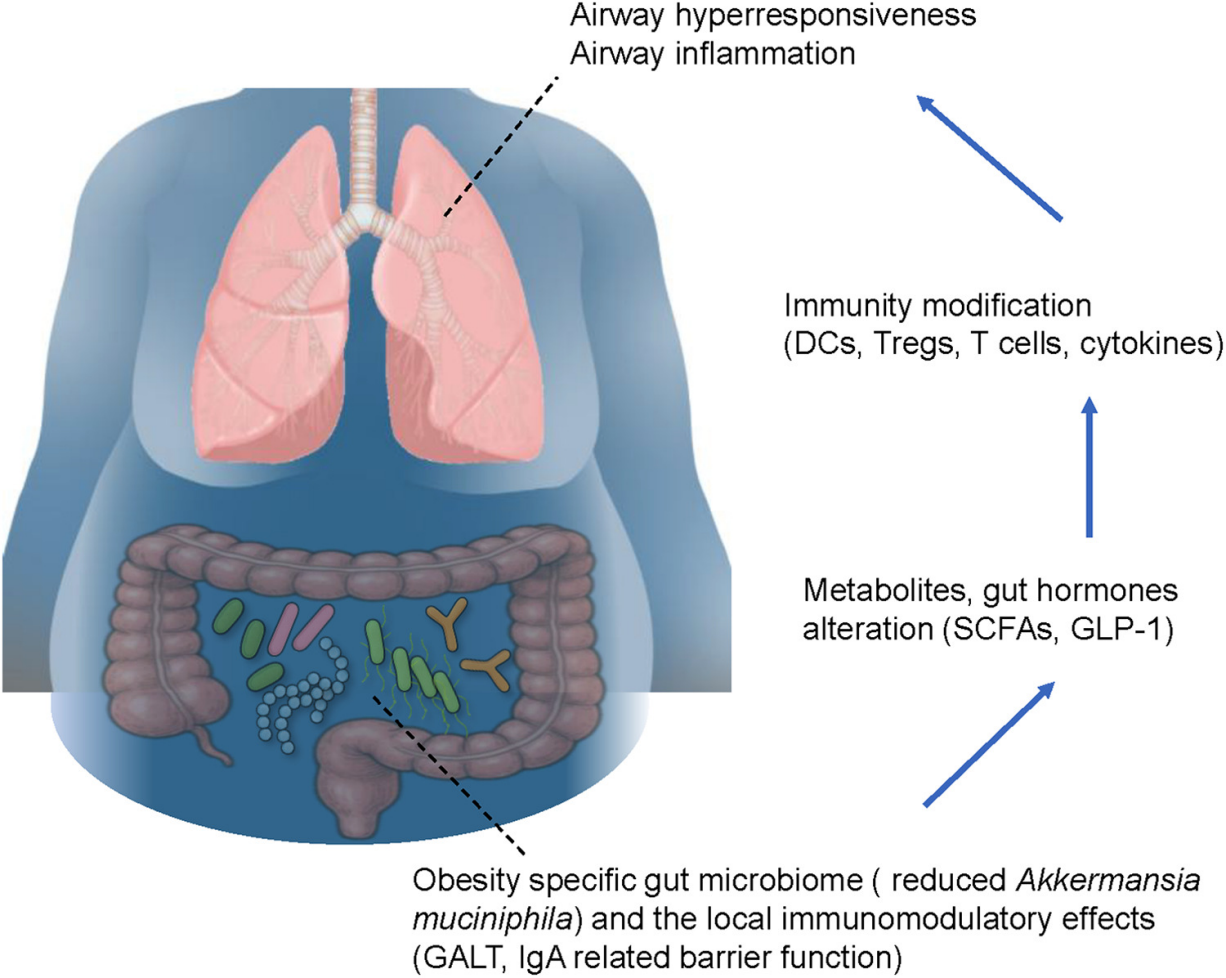
Possible mechanistic basis of the interaction between the gut microbiome and severe asthma with obesity. The obesity-specific gut microbiome is characterized by a reduced proportion of *Akkermansia muciniphila*, which affects the modulation of local immunity via gut-associated lymphoid tissue and immunoglobulin A-related barrier function. These phenomena induce metabolites of the gut microbiome and hormones, such as short chain fatty acids and glucagon-like peptide 1, respectively, leading to modification of lung immunity through dendritic cells, regulatory T cells, T cells, and cytokines. Consequently, airway hyperresponsiveness and airway inflammation are exacerbated in obese asthmatic patients. Blue arrows indicate gut-to-lung signaling pathways. The figure is based on our previous report with partial modification (21). GALT, gut-associated lymphoid tissue; IgA, immunoglobulin A; SCFAs, short-chain fatty acids; GLP-1, glucagon-like peptide 1; DCs, dendritic cells; Tregs, regulatory T cells.

As discussed above, obesity and asthma are both potentially associated with the gut microbiome. Hence, it is understandable that the gut microbiome might affect the severity of obesity-induced asthma. Recently, to clarify the correlation between the gut microbiome and specific asthma phenotypes, 211 gut microbiota taxa were analyzed in a genome-wide association study calculated by Mendelian randomization analysis and sensitivity analysis (85). The study focused on allergic asthma, childhood asthma, and obesity-related asthma, with analysis performed to clarify the causal relationships between the gut microbiome and distinct asthma phenotypes. The results indicated that a higher genetically predicted abundance of the genera *Holdemanella*, *Lachnospiraceae FCS020* group, *Eubacterium xylanophilum* group, *Odoribacter*, and *Lachnospiraceae ND3007* group was associated with an increased risk of obesity-related asthma. In contrast, the genera *Ruminococcaceae UCG010* and *Senegalimassilia* were inversely associated with the risk. At the family level, *Rikenellaceae* and *Pasteurellaceae* were associated with a decreased risk of obesity-related asthma. Furthermore, the bacterial order *NB1-n* showed a positive association with the disease risk, whereas the order *Pasteurellales* demonstrated a protective effect. Other studies also reported that obesity in patients with asthma was associated with elevated levels of proinflammatory molecules in the bloodstream, along with alterations in the gut microbiome. Furthermore, a reduced relative abundance of *Akkermansia muciniphila* has been shown to directly correlate with increased asthma severity, indicating that several specific bacteria affect disease severity in obese asthmatic patients (86). We previously clarified the specific interaction between the gut microbiome and pathophysiology of obesity-induced asthma, such as AHR and neutrophilic airway inflammation, in mice. Briefly, the gut microbiome in lean mice and genetically induced obese mice called db/db mice were different, and ozone-induced AHR and neutrophils in BAL fluid were greater in obese mice than in lean mice. Importantly, depletion of the gut microbiome by a cocktail of antibiotics induced significant recovery of AHR and neutrophilic airway inflammation. Additionally, ozone-induced AHR and neutrophils were greater in germ-free mice with reconstitution of microbes derived from obese mice, than in germ free mice with microbial reconstitution using microbes derived from lean mice, indicating that the gut microbiome itself affects worsening of the severity of asthma with obesity (18). A high-fat diet also has the capacity to manipulate the gut microbiome along with an increase in body weight in mice (20), which augments pulmonary responses following exposure to ozone or HDMs (20, 75, 76). Bariatric surgeries for obesity, including sleeve gastrectomy and Roux-en-Y gastric bypass, decrease body weight and might attenuate AHR and airway inflammation, both of which are involved in the pathophysiology of asthma in obese individuals (87). Importantly, bariatric surgery also affects the gut microbiome, and induces a significant perturbation of the gut microbiome with increased abundance of *Akkermansia muciniphila* at 3 months after the operation, with the effect continuing through 12 months (88). This suggests that the obesity-specific gut microbiome affects the severity of obesity-induced asthma. Additionally, the gut microbiome might be a new therapeutic target in the fight against obesity-induced asthma, and clinical trials of specific interventions to improve perturbations of the gut microbiome are expected in the future (Figure 1). Notably, leptin—one of the important adipokines associated with obese individuals and obesity-related diseases—may also impact the severity of obesity-related asthma through effects on the gut microbiome (89). Indeed, several reports have indicated a strong association between body fat ratio and serum leptin concentration (90, 91). Additionally, leptin itself has the capacity to modify the gut microbiome, which in turn affects body weight in response to dietary fat intake (92). Importantly, db/db mice, which are characterized by leptin receptor deficiency, exhibit a distinct gut microbiome composition compared to wild-type mice. This altered microbiome directly contributes to increased AHR and inflammation, as noted above (18).

### Possible gut microbiome-targeted therapies for obese asthma patients

Various options as gut microbiome-targeted therapies in asthmatic patients with obesity have been suggested, such as fecal microbiome transplantation, probiotics, prebiotics, and the prudent use of antibiotics (1, 21, 93). The purpose of intervention trials for asthma patients with obesity should include endpoints such as exacerbation frequency, pulmonary function, microbiome diversity and serum biomarkers. These trials should also take into account the clinical phenotype of airway inflammation in participants, as well as the type and dosage of the intervention. Several clinical trials have evaluated the efficacy of fecal microbiome transplantation in patients with disease conditions, and it has shown positive results in patients with recurrent *Clostridium difficile* infection and inflammatory bowel syndrome (94–97). In terms of obesity and metabolic syndrome, a fair number of the trials showed that fecal microbiome transplantation did not affect clinical parameters, including BMI, even though it partially improved peripheral insulin sensitivity, suggesting that a short duration of the intervention should be considered (98). Unfortunately, to the best of our knowledge, there is no clinical trial on fecal microbiome transplantation in asthmatic patients with obesity, and future investigation on this is required.

Probiotics are defined as living bacteria that are beneficial for human health. As mentioned above, *Akkermansia muciniphila* might have beneficial effects in obese asthmatic patients (86, 88). Indeed, it was recently reported that heat-killed *Akkermansia muciniphila* strain EB-AMDK19 attenuated HDM-induced AHR, airway inflammation, mucus hyperplasia, and elevation of cytokines and chemokines in mice (99). Other specific bacteria, as mentioned above, might be candidate probiotics for the treatment of asthmatic patients.

Prebiotics, which include dietary fiber, are defined as a group of nutrients that are degraded by gut microbiota (100). Pectin, a fermentable fiber, attenuates HDM-induced AHR and airway inflammation, with elevation of SCFA production, which directly attenuates the pathophysiology of asthma via G protein-coupled receptors (60). We previously reported that worsening of ozone-induced AHR and neutrophilic airway inflammation in obese mice was ameliorated by administration of pectin, but not cellulose, an unfermentable fiber, resulting in elevation of serum SCFAs (18). These data indicated that supplementary administration of a fermentable fiber has beneficial effects on augmented asthma pathophysiology in obese individuals via SCFAs, and that SCFAs might also be candidates in gut microbiome-targeted therapies in obese asthmatic patients. Notably, the effects of SCFAs on AHR and airway inflammation may depend on clinical phenotypes, as suggested by findings from mouse experiments. For example, as mentioned above, SCFAs showed beneficial effects on HDM-induced AHR and eosinophilic airway inflammation in lean mice, as well as on ozone-induced AHR and neutrophilic airway inflammation in obese mice (18, 60). However, SCFAs—particularly propionate—exhibited harmful effects on ozone-induced AHR and neutrophilic airway inflammation in lean mice (16). Although the detailed mechanisms underlying the differential effects of SCFAs on asthma pathophysiology remain unclear, their clinical impact on asthma severity, especially in patients with obesity, should be carefully evaluated. Although induction of resistance and resistant genes should be kept in mind, antibiotics also have a big impact on manipulating the gut microbiome, which might improve asthma pathophysiology in obese individuals. Indeed, antibiotic cocktails reportedly attenuate ozone-induced increases in AHR and airway inflammation in obese mice with depletion of their gut microbiome, as we previously reported (18). Based on clinical experience, macrolide antibiotics are typically prescribed for a long duration in patients with chronic bronchitis and non-tuberculous mycobacterium, and could also be candidate drugs in obese asthmatic patients. Gibson PG et al. reported in a randomized, double-blind, placebo-controlled trial focusing on severe asthmatic patients that azithromycin attenuated exacerbations and led to recovery of the patients’ quality of life (101). We also reported that EM900, another macrolide antibiotic, attenuated exacerbation of HDM-induced airway inflammation in obese mice (76). However, we still do not know whether azithromycin would be effective in attenuating asthma severity in obese individuals. We are currently planning an intervention study of azithromycin for obesity-induced severe asthma to evaluate its efficacy in reducing exacerbations, along with alteration of the gut microbiome and biomarkers, such as cytokines and chemokines (102).

## Conclusion

This review addressed the role of the gut microbiome in severe asthma with obesity. Although careful interpretation is necessary when considering clinical efficacy, as the present review is primarily based on murine data, we have proposed candidate treatments as potential gut microbiome-targeted therapies for this form of asthma. As mentioned previously, several murine studies have demonstrated that fecal microbiota transplantation, probiotics, prebiotics, and antibiotics can ameliorate AHR and airway inflammation in obese asthma models. While FMT remains largely hypothetical as a therapeutic approach in this context, other microbiome-targeted interventions—such as probiotics, prebiotics, and selected antibiotics—may be more feasible for clinical application and warrant further investigation through interventional trials. Due to the shortage of essential data for evaluation of these therapies for asthma with obesity, future clinical trials would be useful.
